# The role of T790M mutation in EGFR-TKI re-challenge for patients with *EGFR*-mutant advanced lung adenocarcinoma

**DOI:** 10.18632/oncotarget.14007

**Published:** 2016-12-17

**Authors:** Qiuyi Zhang, Ee Ke, Feiyu Niu, Wei Deng, Zhihong Chen, Chongrui Xu, Xuchao Zhang, Ning Zhao, Jian Su, Jinji Yang, Honghong Yan, Yilong Wu, Qing Zhou

**Affiliations:** ^1^ Guangdong Lung Cancer Institute, Guangdong General Hospital and Guangdong Academy of Medical Sciences, Guangzhou 510080, Guangdong, PR China

**Keywords:** adenocarcinoma, TKI-free interval, T790M mutation, re-challenge, resistance

## Abstract

Epidermal growth factor receptor (*EGFR*) T790M mutation has shown to be associated with the clinical outcomes of patients after initial EGFR-tyrosine kinase inhibitor (EGFR-TKI) therapy in *EGFR*-mutant advanced non-small cell lung cancer (NSCLC). However, its predictive role in EGFR-TKI re-challenge remains unknown. The present study was aimed to explore the correlation between T790M mutation and any benefits from EGFR-TKI re-challenge. We retrospectively reviewed 922 consecutive patients with *EGFR*-mutant non-small cell lung cancer (NSCLC) patients administered with gefitinib/erlotinib at Guangdong General Hospital. Progression-free survival (PFS), overall survival (OS), objective response rate (ORR) and disease control rate (DCR) were analyzed respectively. In total, 66 *EGFR*-mutant patients with stage IV adenocarcinoma were eligible, of whom 51 underwent re-biopsy upon initial progression. Among them, 18 (35.3%) harbored T790M mutation. No statistical significant differences were seen between T790M-positive and T790M-negative patients in PFS, OS, ORR or DCR. The median PFS, median OS, ORR, and DCR of the overall 66 patients were 2.0 months, 6.8 months, 6.1% and 39.4%, respectively. Good performance status (PS) was found to be independent favorable prognostic factor and long TKI-free interval to be associated with superior PFS. In conclusion, T790M mutation might not predict the clinical outcomes in first-generation EGFR-TKI re-challenge. Based on the poor efficacy from our data, re-challenge of first-generation EGFR-TKIs could not be recommended routinely, but for those with good PS and long TKI-free interval, it might be an alternative option.

## INTRODUCTION

With the dramatic development of translational medicine, therapeutic strategies for non-small cell lung cancer (NSCLC) have entered the era of precision medicine. Epidermal growth factor receptor (EGFR)-tyrosine kinase inhibitors (TKIs), such as gefitinib and erlotinib, have been proven to be effective for advanced NSCLC patients with *EGFR* activating mutations (exon 19 deletion or exon 21 L858R point mutation) compared with traditional platinum-based doublet chemotherapy [1–4]. Unfortunately, patients would inevitably develop acquired resistance after a median progression-free survival (PFS) of 9 to 14 months, despite a dramatic initial response [3, 5]. It is worth mentioning that up to a level of 60% of these patients have been identified with the *EGFR* T790M gatekeeper mutation in exon 20, which was the first described and most frequent molecular alteration involved [6, 7].

However, there is still no standard treatment for patients after acquiring resistance to first-generation EGFR-TKIs at present. For patients with no other targetable biomarkers, chemotherapy may be highly recommended as routine. Although randomized trials with 3rd generation TKIs have confirmed the high effectiveness in the case of T790M mutation positive [8], it is not widely available all around the world. As for mainland China, T790M inhibitors will not be officially approved in the near future, except for the clinical trials participants. Hence, due to limited novel therapeutic strategy upon resistance in clinical practice, there is still a subset of patients who will receive EGFR-TKIs for the second time as salvage treatment after initial failure.

The *EGFR* T790M mutation plays a significant role in EGFR-TKI initiation. Previous studies have demonstrated that the presence of T790M mutation could be a predictive factor for clinical outcomes [9–12]. Su et al. reported that the presence of pre-treatment T790M mutation predicted shorter EGFR-TKI treatment durations [11], and similar results were obtained by Rosell et al. [10]. While Oxnard et al. suggested that patients with post-treatment T790M mutation demonstrated more favorable survivals [9]. Nevertheless, the specific role of T790M mutation in EGFR-TKI re-challenge remains unknown. Based on its predictive role in initial TKI therapy, we hypothesized that patients without T790M mutation would benefit more from and be potential candidates for TKI re-challenge. Thus, to explore the correlation between T790M mutation and any benefits from EGFR-TKI re-challenge, we retrospectively collected the clinical data from consecutive NSCLC patients with *EGFR* activating mutations who were re-challenged with EGFR-TKIs.

## RESULTS

### Patient characteristics

In total of the 922 screened, 66 patients with stage IV lung adenocarcinoma met the inclusion criteria. Among these 66 cases, 51 underwent re-biopsy upon prior progressive disease (PD), and the remaining 15 refused the biopsy. Out of the 51 patients, 18 (35.3%) were found to harbor T790M mutation. The clinical characteristics of these 51 patients are summarized in Table 1, with no significant difference between the T790M-positive and T790M-negative groups in any characteristic. Out of the 51 cases, 11 (21.6%) patients received initial EGFR-TKIs in other hospitals, which did not reveal the exact progression model.

**Table 1:** Patient characteristics between T790M+ and T790M− groups (*n* = 51)

Characteristic	Total (*n* = 51)	T790M+	T790M−	*P*
		(*n* = 18) *n* (%)	(*n* = 33) *n* (%)	
Age (year)				0.171
Median (Range)	58 (30~87)	58 (44~87)	57 (30~82)	
Gender				0.164
Male	19 (37.3)	9 (50.0)	10 (30.3)	
Female	32 (62.7)	9 (50.0)	23 (69.7)	
Smoking status				1.000
Smoker	8 (15.7)	3 (16.7)	5 (15.2)	
Never smoker	43 (84.3)	15 (83.3)	28 (84.8)	
ECOG performance status				1.000
0~1	37 (72.5)	13 (72.2)	24 (72.7)	
≥ 2	14 (27.5)	5 (27.8)	9 (27.3)	
Insertion chemotherapy				0.288
None	11 (21.6)	2 (11.1)	9 (27.3)	
Cytotoxic chemo	40 (78.4)	16 (88.9)	24 (72.7)	
*EGFR* mutation				0.217
Exon 19 deletion	31 (60.8)	13 (72.2)	18 (54.5)	
Exon 21 L858R mutation	20 (39.2)	5 (27.8)	15 (45.5)	
TKI-free interval				0.137
< 3 m	10 (19.6)	6 (33.3)	4 (12.1)	
≥ 3 m	41 (80.4)	12 (66.7)	29 (87.9)	
PFS of initial TKI				0.726
<6 m	11 (21.6)	3 (16.7)	8 (24.2)	
≥ 6 m	40 (78.4)	15 (83.3)	25 (75.8)	
Secondary EGFR-TKIs				0.690
Erlotinib	33 (64.7)	11 (61.1)	22 (66.7)	
Gefitinib	18 (35.3)	7 (38.9)	11 (33.3)	
Line of TKI re-challenge				1.000
Second line	6 (11.8)	2 (11.1)	4 (12.1)	
≥ Second line	45 (88.2)	16 (88.9)	29 (87.9)	
*EGFR* detection assay				0.880
Seq	36 (70.6)	12 (66.6)	24 (72.7)	
ARMS	12 (23.5)	5 (27.8)	7 (21.2)	
Seq+ARMS	3 (5.9)	1 (5.6)	2 (6.1)	
Progression model of initial TKI				0.849
Dramatic	19 (37.2)	7 (38.8)	12 (36.3)	
Local	15 (29.4)	5 (27.8)	10 (30.3)	
Gradual	6 (11.8)	3 (16.7)	3 (9.1)	
Unknown*	11 (21.6)	3 (16.7)	8 (24.2)	

PFS, progression-free survival. Seq, Sanger sequencing. ARMS, Amplification Refractory Mutation System.*These patients were initiated with TKIs in other hospitals.

### Response to initial EGFR-TKIs

During prior gefitinib/erlotinib treatments, out of the 66 patients two cases (3.0%) achieved a complete response (CR), 31 (47.0%) displayed a partial response (PR), 28 (42.4%) maintained a stable disease (SD) and 5 (7.6%) had PD. The median PFS was 10.0 months, with a 95% confidence interval (CI) of 8.4 to 11.6 months. The objective response rate (ORR) and disease control rate (DCR) were 50.0% and 92.4%, respectively.

### Response to secondary EGFR-TKIs

Out of the 66 cases for re-challenge, only 4 (6.1%) patients demonstrated a PR, 22 (33.3%) SD, and 40 (60.6%) patients developed PD. With a median follow-up duration of 67.2 months (range, 17.0 to 329.3 months), the median PFS, overall survival (OS), ORR, and DCR were 2.0 months (95% CI 1.3–2.7), 6.8 months (95% CI 4.7–8.9), 6.1%, and 39.4%, respectively (Table 2).

**Table 2:** Patients’ responses to EGFR-TKI re-challenge

Response	Sequence pattern				Total (*n* = 66)
	G to G (*n* = 13)	E to E (*n* = 12)	G to E (*n* = 33)	E to G (*n* = 8)	
CR (%)	0 (0)	0 (0)	0 (0)	0 (0)	0 (0)
PR (%)	2 (15.4)	1 (8.3)	0 (0)	1 (12.5)	4 (6.1)
SD (%)	5 (38.5)	5 (41.7)	7 (21.2)	5 (62.5)	22 (33.3)
PD (%)	6 (46.1)	6 (50.0)	26 (78.8)	2 (25.0)	40 (66.6)
DCR	53.8%	50.0%	21.2%	75.0%	39.4%
ORR	15.4%	8.3%	0.0%	12.5%	6.1%
Median PFS (95% CI)	3.2 (2.0–4.4)	2.7 (0.5–4.9)	1.2 (0.8–1.6)	2.9 (2.6–3.2)	2.0 (1.3–2.7)
Median OS (95% CI)	5.1 (2.2–8.0)	8.8 (4.1–13.5)	4.1 (2.4–5.8)	9.6 (4.7–14.5)	6.8 (4.7–8.9)

CR, complete response. PR, partial response. SD, stable disease. PD, progression disease. DCR, disease control rate. ORR, objective response rate. PFS, progression-free survival. OS, overall survival. CI, confidence interval G, gefitinib. E, erlotinib.

Furthermore, univariate analysis revealed a favorable PFS for patients with longer TKI-free interval (hazard ratio [HR] 0.48, 95% CI 0.24–0.98, *P* = 0.044) and a prolonged OS for those with good ECOG performance status (PS) (HR 0.34, 95% CI 0.19–0.61, *P* < 0.001), and that patients with insertion chemotherapy tended to exhibit better efficacy although only a borderline significance was observed (HR 0.60, 95% CI 0.34–1.09, *P* = 0.092) (Table 3). As shown in the multivariate analysis, good ECOG PS was found to be the independent favorable prognostic factor (HR 0.32, 95% CI 0.18–0.57, *P* < 0.001) (Table 4). Besides, patients with a longer TKI-free interval tended to exhibit superior PFS though a borderline significance was obtained (HR 0.56, 95% CI 0.31–1.00, *P* = 0.051). No significant differences were found in the remaining characteristics including *EGFR* mutation (19DEL vs. L858R), initial PFS (< 6 m vs. ≥ 6 m) and insertion chemotherapy (Yes vs. No) (data not shown).

**Table 3:** Univariate analyses of PFS and OS between clinical characteristics (*n* = 66)

Characteristics	PFS			OS		
	HR	95% CI	*P*	HR	95% CI	*P*
Gender		0.76–2.07			0.62–1.82	0.815
Male	1.00		0.383	1.00		
Female	1.25			1.07		
Smoking status		0.35–1.38	0.303		0.47–2.12	0.983
Never smoker	1.00			1.00		
Smoker	0.70			0.99		
Age	1.00	0.98–1.02	0.882	1.00	0.98–1.02	0.761
TKI-free interval		0.24–0.98	0.044		0.58–2.67	0.561
< 3 m	1.00			1.00		
≥ 3 m	0.48			1.25		
ECOG PS		0.36–1.03	0.064		0.19–0.61	< 0.001
0~1	1.00			1.00		
≥ 2	0.61			0.34		
EGFR type		0.49–1.36	0.442		0.44–1.28	0.292
Exon 19 deletion	1.00			1.00		
Exon 21 L858R mutation	0.82			0.75		
Initial PFS		0.37–1.25	0.213		0.35–1.28	0.222
< 6 m	1.00			1.00		
≥ 6 m	0.68			0.67		
Insertion chemotherapy		0.34–1.09	0.092		0.57–1.97	0.853
None	1.00			1.00		
Cytotoxic agent	0.60			1.06		

PFS, progression-free survival. OS, overall survival. PS, performance status; HR, hazard ratio. CI, confidence interval.

**Table 4:** Mutivariate analysis of variables for PFS and OS

Characteristics*	PFS			OS		
	HR	95% CI	*P*	HR	95% CI	*P*
TKI-free interval						
< 3 m	1.00					
≥ 3 m	0.56	0.31–1.00	0.051			
ECOG PS						
≥ 2				1.00		
0~1				0.32	0.18–0.57	< 0.001

PFS, progression-free survival. OS, overall survival. CI, confidence interval. HR, hazard ratio. PS, performance status.

* No significant differences were found in the remaining characteristics including *EGFR* type, initial PFS and insertion chemotherapy (data not shown).

### The predictive role of T790M mutation

**Figure 1:**
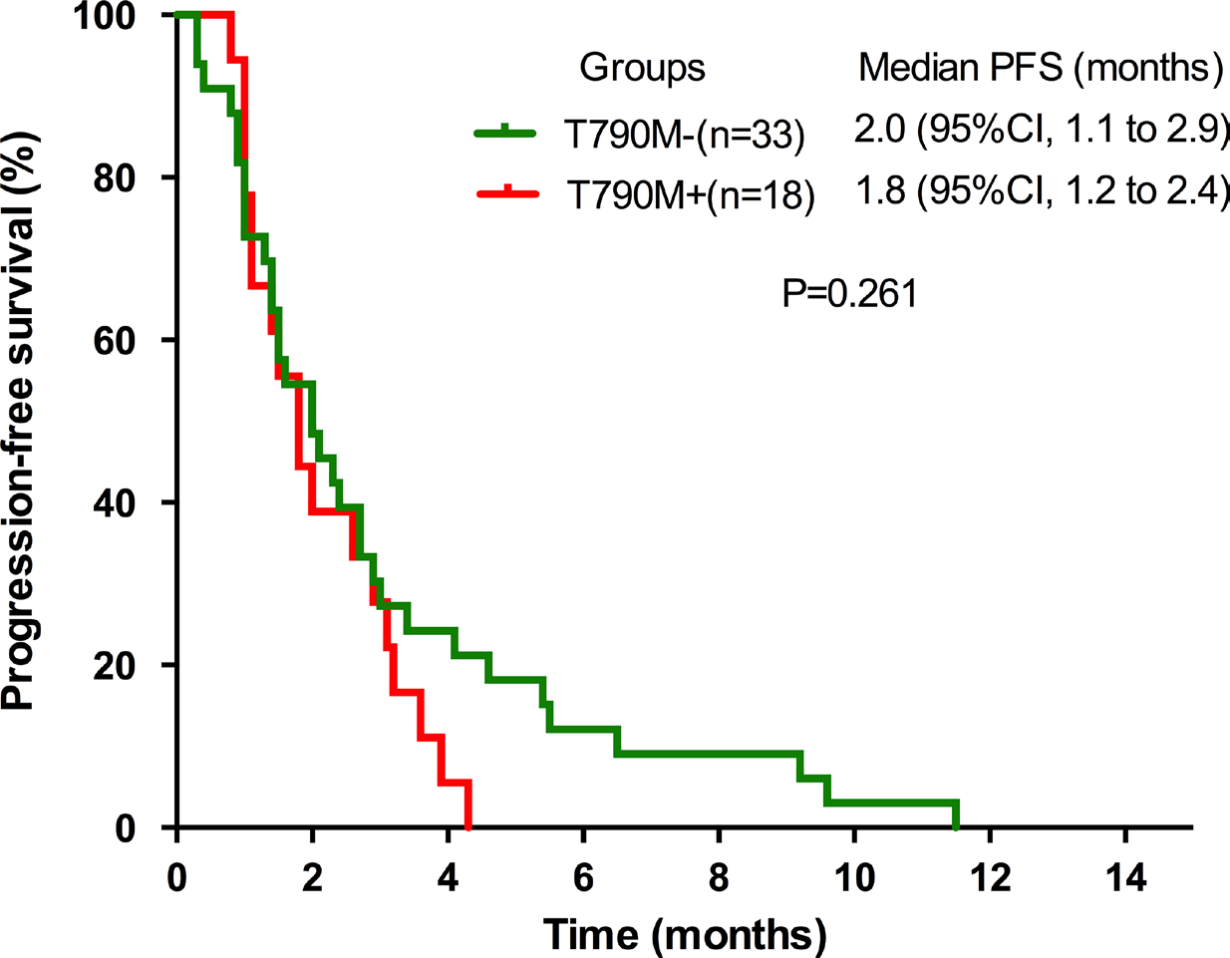
Kaplan-Meier curves of PFS in T790M+ and T790M− groups PFS, progression-free survival.

**Figure 2:**
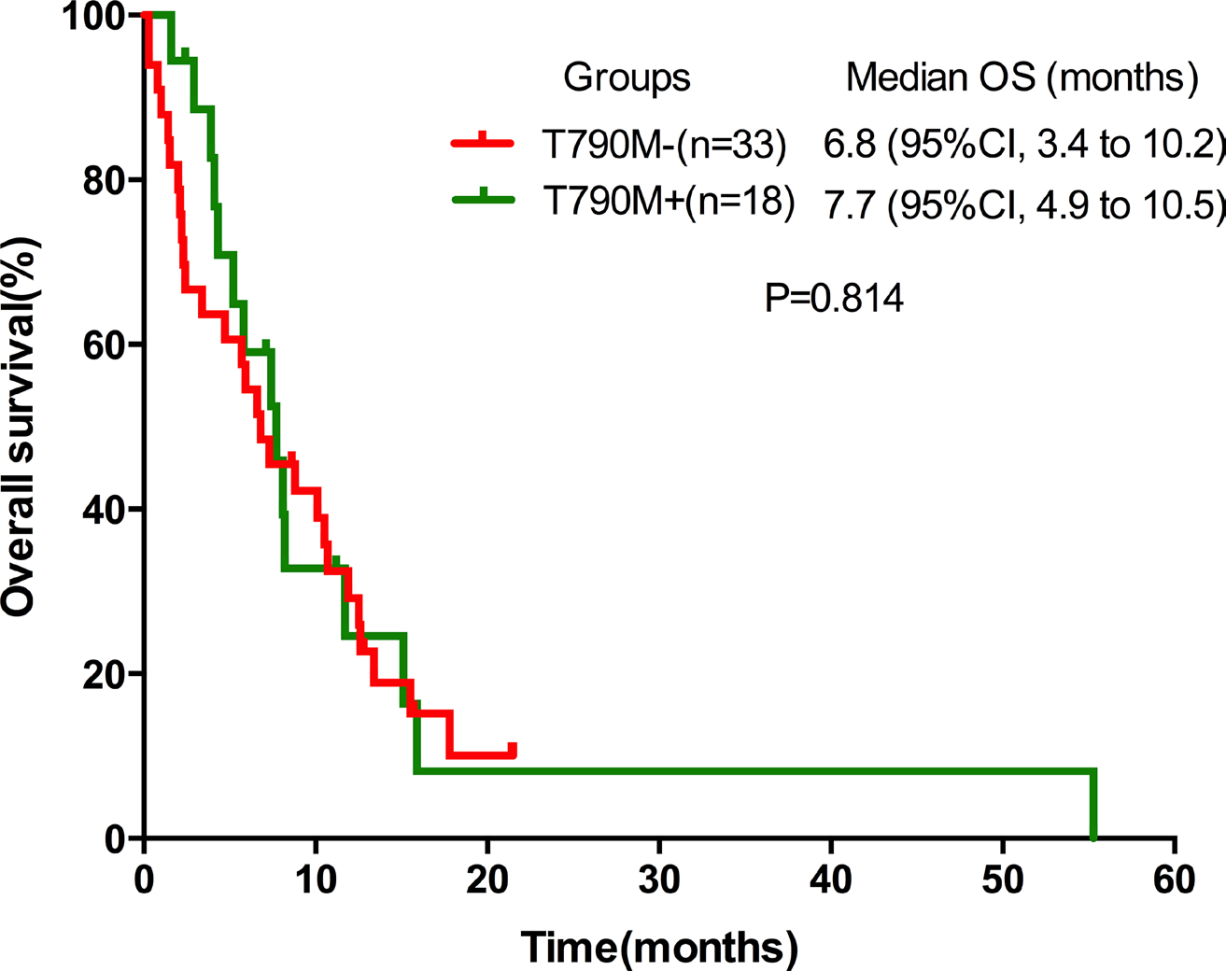
Kaplan-Meier curves of OS in T790M+ and T790M− groups OS, overall survival.

The PFS from the commencement of the secondary EGFR-TKIs did not differ significantly between the T790M-positive and the T790M-negative groups (1.8 months, 95% CI 1.2–2.4 vs. 2.0 months, 95% CI 1.1–2.9, *P* = 0.261) (Figure 1). Similar results were observed for the median OS (7.7 months, 95% CI 4.9–10.5 vs. 6.8 months, 95% CI 3.4–0.2, *P* = 0.814) (Figure 2), ORR (0.0% vs. 12.1%, *P* = 0.284) and DCR (33.3% vs. 36.4%, *P* = 0.829).

## DISCUSSION

To the best of our knowledge, this is the first study to evaluate the relationship between T790M status and the clinical outcomes of patients re-challenged with EGFR-TKIs. Although T790M inhibitor, proved to be highly effective for the T790M-positive patients [13], yet is now only available in America, the Europe, Hongkong, Macau and several other areas. Consequently, it is of great significance to conduct this study due to the lack of therapeutic approaches upon resistance in many other countries. The results from our study showed that the presence of T790M mutation was not associated with a significant difference in PFS, OS, ORR or DCR for first-generation EGFR-TKI re-challenge. Moreover, given the poor efficacy and survival data from our findings, first generation EGFR-TKI re-challenge might not be recommended routinely for resistant patients, regardless of the presence of the T790M mutation. However, another similar report revealed different conclusions. The T790M subgroup analysis of IMPRESS study indicated that for patients with T790M-positive in plasma upon RECIST progression, gefitinib should not be continued when doublet chemotherapy is used in second-line, though merely with 41% maturity of endpoint events. In contrast, for patients with T790M-negative in plasma, gefitinib in combination with doublet chemotherapy may offer clinical benefit [14]. Considering together with our study, the optimal strategy for T790M-negative patients seems to be doublet chemotherapy in combination with EGFR-TKIs, and doublet chemotherapy for T790M-positive patients. Yet, it requires further confirmation in a prospective randomized study.

Several retrospective and phase II studies have evaluated the responses of secondary gefitinib/erlotinib retreatments, and their results seem to favor this strategy [15–19]. However, considering a median PFS of as short as 2.0 months from our data, we do not recommend this strategy routinely, similar to the previous two studies conducted by Costa DB and Lee DH et al. [20, 21]. The PFS of these patients were relatively short probably because 58 (87.9%) of these patients were treated no less than 3 lines upon the secondary EGFR-TKI treatments. The efficacy could be more favorable if this study were conducted prospectively. Consistent with previous reports, our univariate and multivariate analysis suggests that patients with a good PS and a long TKI-free interval could benefit better from TKI re-challenge [15, 22]. In other words, EGFR-TKI re-challenge might be an alternative for this specific subset of patients when no other favorable treatments are available. However, it should be verified by prospective study with large population. In this setting, the ongoing clinical trial CTONG1304 (NCT01933347) in China will bring greater insight into the management of EGFR-TKI re-challenge. Even though, it is still reasonable to perform a re-biopsy upon progression and the best resistance-conquering solution is to present a targeted therapy based on individual molecular profile.

An advantage of the present study is that it was conducted, based on the electronic medical record database at Guangdong Lung Cancer Institute (GLCI). Despite the retrospective nature of our study, the results are reliable. The objective tumor responses were evaluated strictly according to the RECIST criteria. Moreover, our center has established its own follow-up protocol since 2004, and tumor assessments for patients treated with EGFR-TKIs were strictly performed following the protocol at every 8 weeks in clinical practice (*n* = 60) and every 6 weeks in clinical trials (*n* = 6). Among the six trial patients, one harbored T790M mutation, while three did not, and the remaining two didn't undergo re-biopsy.

Still, it is a pity to say that there are some limitations in our study. One is that the *EGFR* detections were performed by direct sequencing before the year 2012 or ARMS assay since 2012, because ARMS assay was not officially approved in our center until when the solid evidence of higher sensitivity of ARMS compared with sequencing was reported [23]. Therefore, the meaning of positivity of T790M mutation is different in terms of quantity of T790M mutation positive cancer cells in the present study. Among the 51 patients who underwent re-biopsy upon initial progression, only 18 (35.3%) of them were found to harbor T790M mutation, which was much lower than previously reported. The primary explanation was that 36 (70.6%) specimens were genotyped using direct sequencing with much lower sensitivity than ARMS. But it indicated no statistical differences in the results obtained between the two methods from our data. Additionally, due to the long washout period of the data collection, the evaluations were assessed by Response Evaluation Criteria in Solid Tumors (RECIST) version 1.0 (before 2009) or 1.1 (since 2009), leading to certain inconsistence in our study. Lastly, the small population sample size could have caused the lack of significant statistically differences.

In addition to the clinical analysis, our team is now working on the experiment design, and the related experiments on these clinical samples should be carried out after comprehensive consideration. We believe the data would be available in due course. All together, our study indicates that the *EGFR* T790M mutation may not be associated with clinical outcomes of first-generation EGFR-TKI re-challenge for *EGFR*-mutant advanced lung adenocarcinoma patients. In addiction, EGFR-TKI re-challenge might not be recommended routinely after initial resistance to TKIs, but rather as an alternative option for the specific patients with a good PS and a long TKI-free interval. Most importantly, prospective randomized studies based on biomarkers are urgently warranted in the future.

## MATERIALS AND METHODS

### Patient eligibility

**Figure 3:**
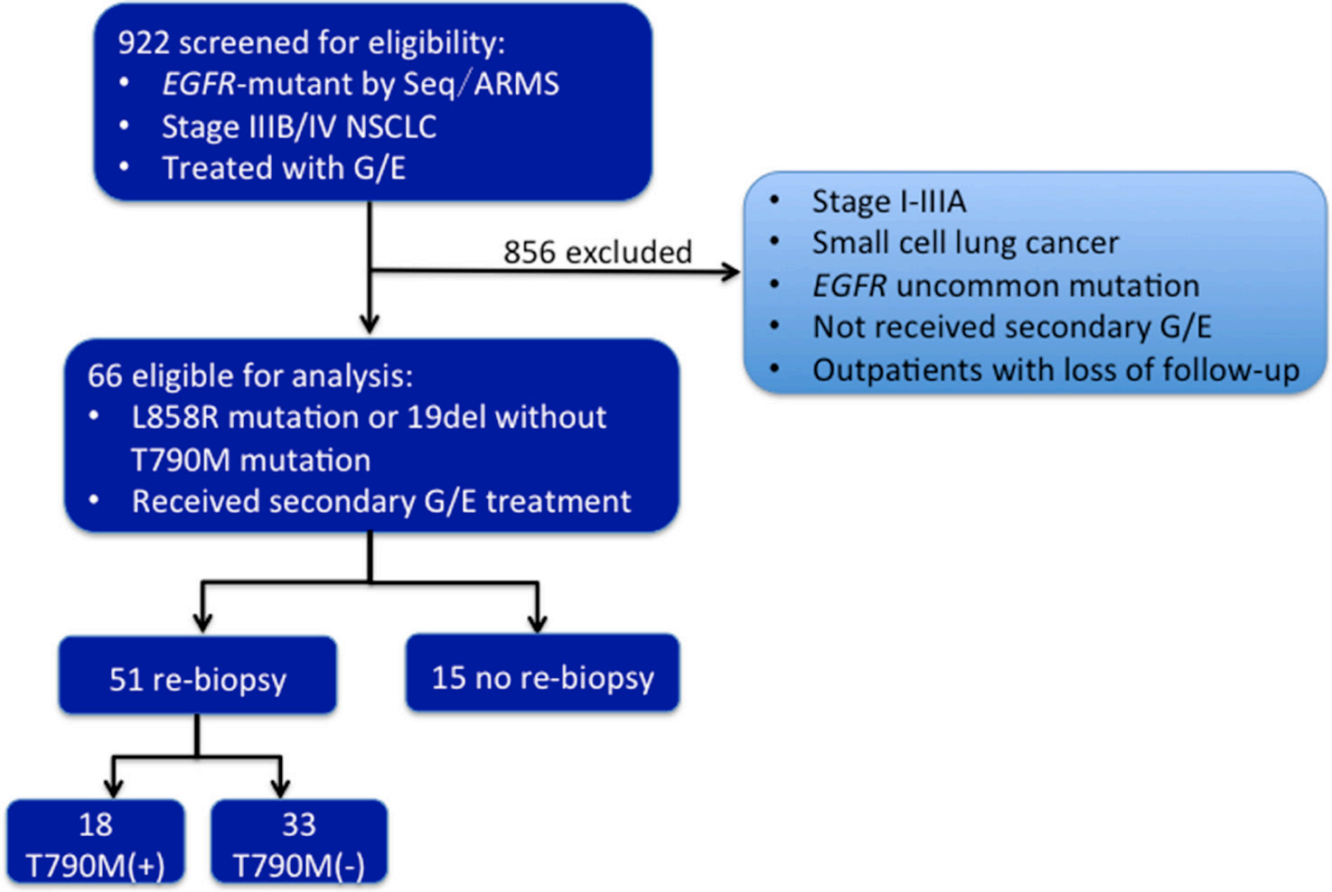
The flowchart of screening for the patient eligibility G, gefitinib. E, erlotinib. Seq, direct sequencing. ARMS, amplification refractory mutation system.

Based on the electronic medical record database at the GLCI, the clinical courses of 922 consecutive *EGFR*-mutant NSCLC patients who received gefitinib or erlotinib from December 2004 to December 2014 were reviewed retrospectively. GLCI was certified by Good Clinical Practice, and informed consent of data collection was obtained from each patient. Among the 922 patients, we identified 66 advanced NSCLC patients with *EGFR* activating mutations (without de novo T790M mutation) re-challenged with gefitinib or erlotinib, including 51 who underwent tumor re-biopsy upon the initial EGFR-TKI failure before the next-line treatment. The screening of the patient eligibility is showed in Figure 3.

### Assessments of the response

Patients received secondary gefitinib/erlotinib in clinical trials or practice and the assessments were performed as described previously by Zhou et al. [24]. The smoking status, medical history and clinical characteristics of each patient were well documented. The radiographic response to EGFR-TKI treatment was assessed every 6 weeks (in clinical trials) or every 8 weeks (in clinical practice), or whenever disease progression was suspected. For evaluation, each patient underwent physical examination, laboratory tests and computed tomography scans covering the chest and upper abdomen. When bone or brain metastasis was suspected, radionuclide bone scans or magnetic resonance imaging was performed. The objective response was determined according to the RECIST guidelines version 1.0 (from 2004 to 2009) or version 1.1 (from 2009 to 2014). The last follow up was May 14, 2015.

### Identification of the EGFR mutations

Genomic DNA from 66 pre-treatment and 51 post-treatment tissue samples were genotyped for the *EGFR* mutation (exons, 18–21) by Sanger sequencing before November 2011 or amplification refractory mutation system assay (ARMS, DxS, Manchester, United Kingdom) after November 2011 as described previously [24]. DNA extraction was performed using the DNeasy Blood and Tissue Kit (No. 69504; Qiagen, Valencia, CA, USA).

### Statistical analysis

PFS was calculated from the initiation of secondary gefitinib/erlotinib to disease progression or to death (of any cause), according to the RECIST criteria. OS was determined from the commencement of secondary gefitinib/erlotinib to death (of any cause), with living patients censored on the date of the last follow-up. Both PFS and OS were estimated using the Kaplan-Meier method and compared between subgroups using the log-rank test. Comparisons of the ORR and the DCR as well as the characteristics of the two groups were carried out using the χ^2^ test, independent *T* test or the Fisher's exact test as appropriate. To evaluate the independent predictive factors for PFS and OS, univariate and multivariate analyses were conducted using Cox proportional hazards regression model (Enter and Forward LR method, respectively). Two-sided values of *P* < .05 were considered statistically significant. Statistical analysis was performed using IBM SPSS version 22.0 software (New York, USA).
